# Approach for growth of high-quality and large protein crystals

**DOI:** 10.1107/S090904951003445X

**Published:** 2010-11-05

**Authors:** Hiroyoshi Matsumura, Shigeru Sugiyama, Mika Hirose, Keisuke Kakinouchi, Mihoko Maruyama, Ryota Murai, Hiroaki Adachi, Kazufumi Takano, Satoshi Murakami, Yusuke Mori, Tsuyoshi Inoue

**Affiliations:** a Graduate School of Engineering, Osaka University, Suita, Osaka 565-0871, Japan; b JST, Japan; c SOSHO Inc., Osaka 541-0053, Japan; d Graduate School of Bioscience and Biotechnology, Tokyo Institute of Technology, Nagatsuta, Midori-ku, Yokohama 226-8501, Japan

**Keywords:** semi-solid agarose gels, top-seeded solution growth, large-scale hanging-drop method, X-ray crystallography, neutron crystallography

## Abstract

Three crystallization methods for growing large high-quality protein crystals, *i.e.* crystallization in the presence of a semi-solid agarose gel, top-seeded solution growth (TSSG) and a large-scale hanging-drop method, have previously been presented. In this study the effectiveness of crystallization in the presence of a semi-solid agarose gel has been further evaluated by crystallizing additional proteins in the presence of 2.0% (*w*/*v*) agarose gel, resulting in complete gelification with high mechanical strength. In TSSG the seed crystals are hung by a seed holder protruding from the top of the growth vessel to prevent polycrystallization. In the large-scale hanging-drop method, a cut pipette tip was used to maintain large-scale droplets consisting of protein–precipitant solution. Here a novel crystallization method that combines TSSG and the large-scale hanging-drop method is reported. A large and single crystal of lysozyme was obtained by this method.

## Abstract 2

Three crystallization methods, including crystallization in the presence of a semi-solid agarose gel, top-seeded solution growth (TSSG) and a large-scale hanging-drop method, have previously been presented. In this study, crystallization has been further evaluated in the presence of a semi-solid agarose gel by crystallizing additional proteins. A novel crystallization method combining TSSG and the large-scale hanging-drop method has also been developed.

## 1. Introduction

The three-dimensional structures of proteins are widely utilized to interpret the biological phenomena involved in proteins and to develop new pharmaceutical products, since the structures demonstrate how the proteins function and how they interact with specific molecules. X-ray and neutron structure analyses are the most popular techniques for determining the three-dimensional structure of proteins. However, the production of large high-quality protein crystals remains a bottleneck in X-ray and neutron crystallography.

To date, crystallographers have developed many protein crystallization methods, such as microbatch, dialysis, counter-diffusion and hanging-drop, as well as sitting-drop vapor diffusion methods (Chayen, 1996 ▶, 1999 ▶; D’Arcy *et al.*, 1996 ▶; McPherson, 1982 ▶, 1999 ▶). Although each of these current techniques has its merits, limitations still remain. To overcome these limitations we previously presented novel crystallization methods, including crystallization in the presence of the semi-solid agarose gel (Hasenaka *et al.*, 2009 ▶; Sugiyama *et al.*, 2009*a* ▶,*b* ▶; Tanabe *et al.*, 2009 ▶), top-seeded solution growth (TSSG) (Shimizu *et al.*, 2009 ▶, 2010 ▶) and a large-scale hanging-drop method (Kakinouchi *et al.*, 2010 ▶), to grow large high-quality protein crystals. Here we further evaluate crystallization in the presence of the semi-solid agarose gel, and develop a new method by combining TSSG and the large-scale hanging-drop method.

## 2. Crystallization in semi-solid agarose gel

### 2.1. Introduction

Agarose gel media is known to prevent natural convection and crystal sedimentation, improving the internal order of the protein crystal (Garcia-Ruiz *et al.*, 2001 ▶; Lorber *et al.*, 1999 ▶). The agarose gel is economical, easy to prepare and suitable over a wide range of pH (3.0 to 9.0). Therefore, several proteins have been crystallized in the presence of agarose gel to improve the crystal quality. However, most of these studies demonstrated crystallization in agarose at concentrations between 0.0 and 0.6% (*w*/*v*) (Willaert *et al.*, 2005 ▶), where agarose exhibits low mechanical strength.

Owing to the mechanical properties, we anticipated that such gel-grown crystals would tolerate environmental perturbations such as evaporation, temperature change and osmotic shock. We previously reported protein crystallization in the presence of a semi-solid agarose gel (Sugiyama *et al.*, 2009*a* ▶), and the gel around the crystals and the gel-grown crystals can be processed by a femtosecond laser for subsequent X-ray diffraction experiments (Hasenaka *et al.*, 2009 ▶; Sugiyama *et al.*, 2009*b* ▶). Furthermore, we have also discovered that the nucleation of proteins is promoted by agarose greater than 1.0% (*w*/*v*) (Tanabe *et al.*, 2009 ▶). So far, four proteins (thaumatin, elastase, glucose isomerase and lysozyme) have been crystallized in the presence of the semi-solid 1.5 to 2.0% (*w*/*v*) agarose gel (Sugiyama *et al.*, 2009*b* ▶). Still, only these four proteins were confirmed to be crystallizable using this method, and they were crystallized at pHs ranging from 4.5 to 8.0 using salts or organic solvents as precipitants but not polyethylene glycol (PEG). Here, we crystallize additional proteins (xylanase, insulin and glucose isomerase) under different crystallization conditions to evaluate the effectiveness of the crystallization method.

### 2.2. Crystallization in the presence of the semi-solid agarose gel

Xylanase from *Trichoderma reesei* (Hampton Research), bovine insulin (Sigma-Aldrich) and glucose isomerase from *Streptomyces rubiginosus* (Hampton Research) were purchased and used without further purification. Xylanase dissolved in 43% (*w*/*v*) glycerol and 0.18 *M* Na/K phosphate (pH 7) was diluted in 100 m*M* Tris-HCl (pH 8.0) at a concentration of 20 mg ml^−1^. Powdered insulin was dissolved in the corresponding buffers (Table 1 ▶) to the required concentrations (15 and 10 mg ml^−1^). Glucose isomerase dissolved in 6 m*M* Tris-HCl (pH 7.0), 0.91 *M* ammonium sulfate and 1 m*M* magnesium sulfate was diluted in 6 m*M* Tris-HCl (pH 7.0) at a concentration of 1 mg ml^−1^. These protein solutions were subsequently passed through a 0.45 µm pore filter. Sea plaque agarose (agarose SP) purchased from Lonza was used in a series of the experiments. Six percent (*w*/*v*) agarose solutions were first prepared by slowly stirring agarose powder in water at room temperature and melting at 373 K. The 6.0% (*w*/*v*) agarose solutions were then stored at 277 K. Before setting up the crystallization trials, the gels were remelted at 368 K and kept as liquid at 308 K.

All crystallization experiments were performed by the batch method using 96-well micro-batch plates (Hampton Research) using the micro-batch method. The crystallization solutions were prepared by mixing equal volumes (2.0 µl) of protein solution, precipitating agent and agarose solution. We selected a final concentration for the agarose of 2.0% (*w*/*v*), because a solution at a lower agarose concentration range [*e.g.* 0 to 1.0% (*w*/*v*)] would not exhibit high mechanical strength, and high viscosities of 3.0 to 6.0% (*w*/*v*) agarose usually hampers the creation of a homogeneous mixture of protein and agarose. The mixtures were immediately loaded onto the micro-batch plates. The crystallization conditions for the xylanase, insulin and glucose isomerase used in this study are summarized in Table 1 ▶. The drops were completely gelled in 30 min. All crystallizations were carried out at 293 K.

**Figure 1:**
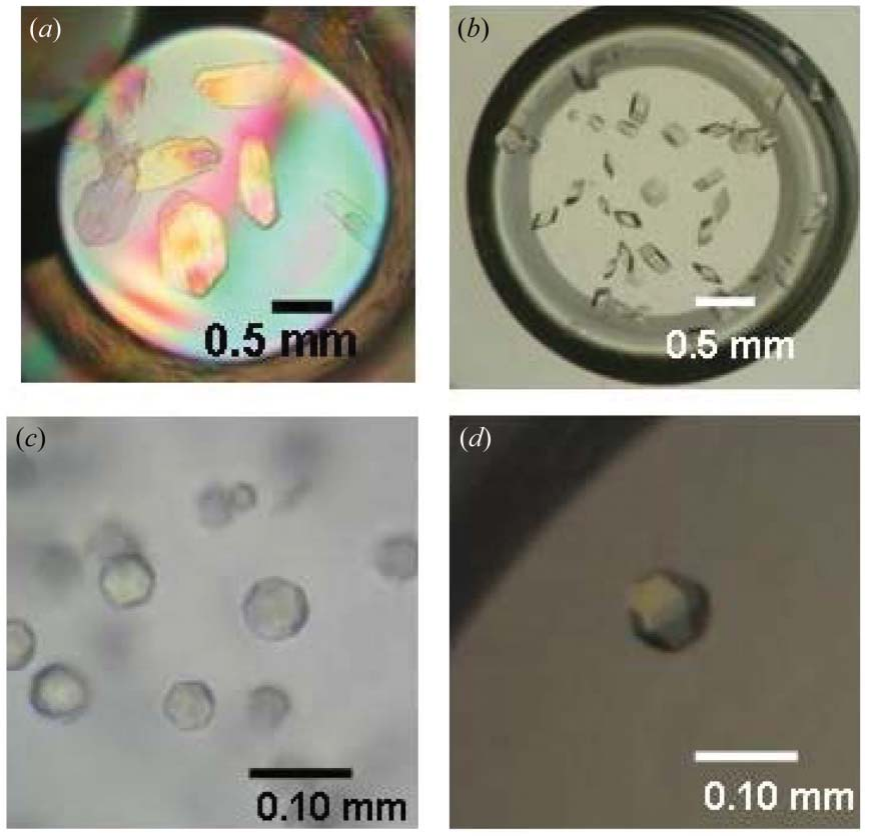
Photographs of protein crystals grown in semi-solid agarose gel. (*a*) Xylanase. (*b*) Insulin at neutral pH. (*c*) Insulin at higher pH. (*d*) Glucose isomerase.

In the semi-solid 2.0% (*w*/*v*) agarose gel, xylanase crystals, two types of insulin crystals and glucose isomerase crystals were successfully obtained within a week (Fig. 1 ▶). The insulin was crystallized at pH >9.0 although the proteins have been crystallized at pH values ranging from 4.5 to 8.0 so far. The glucose isomerase was crystallized using polyethylene glycol, which is a commonly used precipitant for protein crystallization. These results suggest that the protein crystallization in the semi-solid agarose gel may be suitable for general use. Because the crystals are surrounded by the semi-solid gel, this technique enables contactless manipulation of the fragile crystals, which in turn leads to high-quality X-ray diffraction data. Indeed, the diffraction data from the xylanase and insulin crystals grown in semi-solid gel appeared good enough to determine the structures (details will be described elsewhere). Thus, protein crystals grown in semi-solid gel are expected to have some advantages for X-ray crystallography.

## 3. Large crystal growth

### 3.1. Introduction

Neutron protein crystallography is capable of visualizing hydrogen atoms and hydration water molecules to yield a more precise understanding of the protein structure. However, very large crystals (*e.g.* 1–10 mm^3^) (Myles, 2006 ▶) of the target proteins are required to determine the structures by neutron crystallography. To date, several crystallization techniques for growing large protein crystals have been developed, including macroseeding, the two-liquid system (Adachi *et al.*, 2002 ▶), the slow-cooling method (Matsumura *et al.*, 2008 ▶) and the floating-and-stirring technique (Adachi *et al.*, 2004 ▶). Still, several obstacles remain in these methods, in part because protein crystals tend to be polycrystallized.

To prevent polycrystallization during growth of large protein crystals we previously applied TSSG for protein crystallization. TSSG enabled us to grow large crystals of human immunodeficiency virus 1 (HIV-1) protease-inhibitor complex and hen egg-white lysozyme (HEWL) (Shimizu *et al.*, 2009 ▶, 2010 ▶). The high-resolution (1.9 Å) neutron structure has been determined by using a crystal of HIV-1 protease-inhibitor complex grown by TSSG (Adachi *et al.*, 2009 ▶). Although TSSG effectively prevents polycrystallization to obtain a single large protein crystal, this method requires a large amount of protein and precipitating agents (*e.g.* 1 ml). The large-scale hanging-drop method was developed to resolve these issues (Kakinouchi *et al.*, 2010 ▶). This simple method can also be applied to the vapor diffusion method, permitting the concentration of the precipitant agent to be adjusted by vapor diffusion. Furthermore, 200 µl of solution consisting of protein and precipitating agents is sufficient to apply this method. However, unlike TSSG, we must frequently remove spontaneously nucleated crystals; otherwise the seed crystal will be polycrystallized. Here we combined the large-scale hanging-drop method with TSSG to overcome the limitations.

### 3.2. Combination of the large-scale hanging-drop method and TSSG

**Figure 2:**
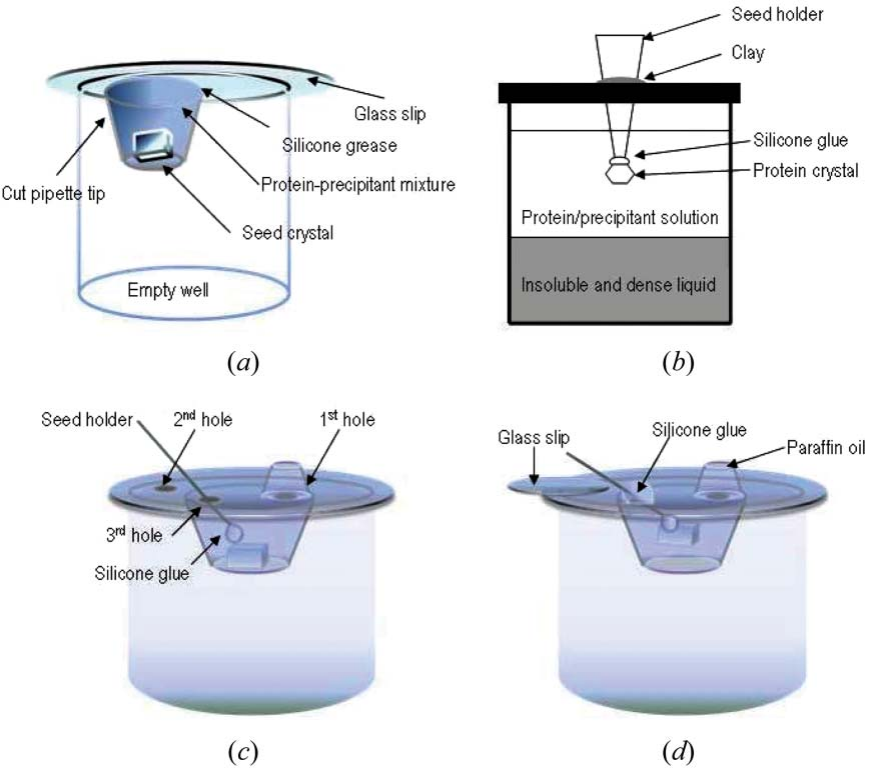
(*a*) Schematic illustration of the crystallization set-up for TSSG. (*b*) Schematic diagram of the protocol of the large-scale hanging-drop method. (*c*) Schematic diagram of the combined method. (*d*) Set-up of this method. The seed holder was lifted up from the interface with the seed crystal after the glue solidified.

We will briefly describe TSSG and the large-scale hanging-drop method. Fig. 2(*a*) ▶ schematically illustrates the crystallization set-up for TSSG (Shimizu *et al.*, 2009 ▶). The protein–precipitant mixture (*e.g.* 1 ml) was placed onto an insoluble and highly dense liquid (Fluorinert). The seed crystal is hung with silicone glue (GE Toshiba Silicones, Japan) in a seed holder protruding from the top of the growth vessel. In this method a single crystal of HEWL grew to a maximum size of 2.6 × 2.1 × 1.4 mm and a single crystal of HIV-1 protease-inhibitor KNI-272 complex grew to a maximum size of 3.2 × 1.9 × 0.6 mm.

Fig. 2(*b*) ▶ schematically illustrates the set-up for the large-scale hanging-drop method (Kakinouchi *et al.*, 2010 ▶). The seed crystals grow at the vapor–liquid interface of the large hanging drop. A cut 1 ml pipette tip with grease at one end can maintain 200 µl of the protein–precipitant mixture. This method prevents protein crystals from adhering to the growth vessel in the same manner as in the hanging-drop method, the two-liquid system (Adachi *et al.*, 2004 ▶) and containerless techniques (Chayen, 1996 ▶; Lorber, 1996 ▶). In addition, spontaneously nucleated crystals are accumulated at the vapor–liquid interface, where they can be removed by nylon loops. In this method a crystal of peroxiredoxin from *Aeropyrum pernix* K1 grew to a size of 2.5 × 2.5 × 0.5 mm (3.1 mm^3^), and a crystal of HEWL grew to a size of 1.0 × 0.5 × 0.5 mm (Kakinouchi *et al.*, 2010 ▶).

**Figure 3:**
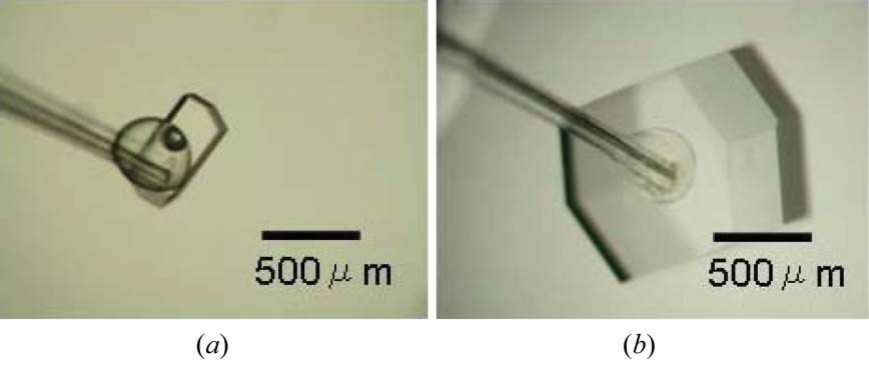
Crystal growth results with the improved large-scale hanging-drop method. (*a*) Seed crystals of HEWL crystal lifted by the seed holder. (*b*) Grown crystal with a size of 1.0 × 1.0 × 0.5 mm after 12 days.

Whereas the methods described above are effective for growing large crystals of proteins, several limitations still remain. As discussed above, care has to be taken to prevent polycrystallization, although the large-scale hanging-drop method enables easy removal of spontaneously nucleated crystals. In another limitation, TSSG requires a large amount of protein (*e.g.* 1 ml) in our system. TSSG is basically a batch method, and it is difficult to frequently adjust the concentration of the precipitating agent, since the cap has to be uncovered to exchange the protein–precipitant mixture and the cap also plays a role in fixing the seed crystal. To overcome these limitations we developed the improved method reported here by combining the large-scale hanging-drop method with TSSG. In this novel method, the three 3 mm holes are made in the glass slips with a diamond cutter (Fig. 2*c* ▶). The first hole is used to exchange the protein–precipitant mixture, the second hole is for pressure control during the protein–precipitant exchange, and the third hole is used to fix the seed holder by silicone glue. HEWL was tested to assess crystal growth using this method. As seed crystals, the HEWL was crystallized at a protein concentration of 30 mg ml^−1^ using the batch method in 0.5 *M* sodium chloride with 0.1 *M* sodium acetate buffer (pH 4.5) at 293 K. As reported previously (Kakinouchi *et al.*, 2010 ▶), a cut 1 ml pipette tip with grease at one end was attached to the glass slip using silicone grease (Dow Corning Toray) in such a way as to surround the droplet, including the seed crystals. 200 µl of the protein–precipitant mixture containing 20 mg ml^−1^ HEWL in 0.5 *M* sodium chloride with 0.1 *M* sodium acetate buffer (pH 4.5) was then injected into the cut tip, and the glass slips were inverted to permit sealing onto the empty well plate (Hampton Research). A cut 200 µl pipette tip with grease at one end was attached to the glass slip using silicone grease (Dow Corning Toray) in such a way as to surround the first hole (Fig. 2*c* ▶). A glass rod of diameter 0.1 mm was prepared as a seed holder. The seed holder with silicone glue was passed through the second hole and was kept in contact with the seed crystal for 24 h to solidify the glue (GE Toshiba Silicones, Japan) (Fig. 2*c* ▶). After the glue solidified, the seed holder was lifted up from the interface with the seed crystal, and the seed holder was fixed to the glass slip by silicone glue (Fig. 2*d* ▶). The first, second and third holes are closed by paraffin oil, glass slips and silicone glue, respectively. Twice a week 100 µl of the protein–precipitant mixture was exchanged using a pipette through the first hole. In this method the seed crystals of HEWL (of size 0.4 × 0.2 × 0.2 mm; Fig. 3*a* ▶) typically grew to a size of 1.0 × 1.0 × 0.5 mm after 12 days (Fig. 3*b* ▶), and the method appeared to prevent polycrystallization. Thus, the combined method enables easy exchange of the protein–precipitant mixture, and the removal of spontaneously nucleated crystals is not always necessary since the spontaneously nucleated crystals are accumulated at the liquid–vapor interface. Thus, this relatively simple method has the potential for general use for growing large crystals of various proteins.

## Figures and Tables

**Table 1:** Crystallization conditions in the presence of 2.0% (*w*/*v*) agarose gel

	Proteins (source)			
	Xylanase (*T. reesei*)	Insulin (bovine pancreas)	Insulin (bovine pancreas)	Glucose isomerase (*Streptomyces rubiginosus*)
Protein concentration (mg ml^−1^)	20	15	10	1
Precipitant	1.0 *M* Na/K phosphate	0.2 *M* Na_2_HPO_4_, pH 9.0	0.1 *M* Na_2_HPO_4_, pH 9.0	5% (*w*/*v*) PEG6000, 0.1 *M* CaCl_2_
Buffer	100 m*M* Tris-HCl (pH 8.0)	10 m*M* Tris-HCl, pH 3.0	50 m*M* Glycine-NaOH, pH 10.0	0.1 *M* Tris-HCl (pH 7.0)

## References

[bb2] Adachi, H., Takano, K., Matsumura, H., Inoue, T., Mori, Y. & Sasaki, T. (2004). *J. Synchrotron Rad.* **11**, 121–124. 10.1107/s0909049503026980 14646151

[bb1] Adachi, H., Watanabe, T., Yoshimura, M., Mori, Y. & Sasaki, T. (2002). *Jpn. J. Appl. Phys.* **41**, L726–L728.

[bb3] Adachi, M., Ohhara, T., Kurihara, K., Tamada, T., Honjo, E., Okazaki, N., Arai, S., Shoyama, Y., Kimura, K., Matsumura, H., Sugiyama, S., Adachi, H., Takano, K., Mori, Y., Hidaka, K., Kimura, T., Hayashi, Y., Kiso, Y. & Kuroki, R. (2009). *Proc. Natl. Acad. Sci. USA*, **106**, 4641–4646. 10.1073/pnas.0809400106 PMC2660780 19273847

[bb4] Chayen, N. E. (1996). *Protein Eng.* **9**, 927–929. 10.1093/protein/9.10.927 8931133

[bb5] Chayen, N. E. (1999). *J. Cryst. Growth*, **198**–**199**, 649–655.

[bb6] D’Arcy, A., Elmore, C., Stihle, M. & Johnston, J. E. (1996). *J. Cryst. Growth*, **168**, 175–180.

[bb7] Garcia-Ruiz, J. M., Novella, M. L., Moreno, R. & Gavira, J. A. (2001). *J. Cryst. Growth*, **232**, 165–172.

[bb8] Hasenaka, H., Sugiyama, S., Hirose, M., Shimizu, N., Kitatani, T., Hasenaka, H., Takahashi, Y., Adachi, H., Takano, K., Murakami, S., Inoue, T., Mori, Y. & Matsumura, H. (2009). *J. Cryst. Growth*, **312**, 73–78.

[bb9] Kakinouchi, K., Nakamura, T., Tamada, T., Adachi, H., Sugiyama, S., Maruyama, M., Takahashi, Y., Takano, K., Murakami, S., Inoue, T., Kuroki, R., Mori, Y. & Matsumura, H. (2010). *J. Appl. Cryst.* **43**, 937–939.

[bb10] Lorber, B. G. R. (1996). *J. Cryst. Growth*, **168**, 204–215.

[bb11] Lorber, B., Sauter, C., Robert, M. C., Capelle, B. & Giegé, R. (1999). *Acta Cryst.* D**55**, 1491–1494. 10.1107/s0907444999008902 10489443

[bb13] McPherson, A. (1982). *The Preparation and Analysis of Protein Crystals* New York: Wiley.

[bb14] McPherson, A. (1999). *Crystallization of Biological Macromolecules.* New York: Cold Spring Harbor Laboratory Press.

[bb12] Matsumura, H., Adachi, M., Sugiyama, S., Okada, S., Yamakami, M., Tamada, T., Hidaka, K., Hayashi, Y., Kimura, T., Kiso, Y., Kitatani, T., Maki, S., Yoshikawa, H. Y., Adachi, H., Takano, K., Murakami, S., Inoue, T., Kuroki, R. & Mori, Y. (2008). *Acta Cryst.* F**64**, 1003–1006. 10.1107/S1744309108029679 PMC2581681 18997326

[bb15] Myles, D. A. (2006). *Curr. Opin. Struct. Biol.* **16**, 630–637. 10.1016/j.sbi.2006.08.010 16963258

[bb16] Shimizu, N., Sugiyama, S., Maruyama, M., Yoshikawa, H. Y., Takahashi, Y., Adachi, H., Takano, K., Murakami, S., Inoue, T., Matsumura, H. & Mori, Y. (2009). *Cryst. Growth Des.* **9**, 5227–5232.

[bb17] Shimizu, N., Sugiyama, S., Maruyama, M., Yoshikawa, H. Y., Takahashi, Y., Adachi, M., Tamada, T., Hidaka, K., Kimura, K., Kiso, Y., Adachi, H., Takano, K., Murakami, S., Inoue, T., Kuroki, R., Mori, Y. & Matsumura, H. (2010). *Cryst. Growth Des.* **10**, 2990–2994.

[bb18] Sugiyama, S., Hasenaka, H., Hirose, M., Shimizu, N., Kitatani, T., Hasenaka, H., Takahashi, Y., Adachi, H., Takano, K., Murakami, S., Inoue, T., Mori, Y. & Matsumura, H. (2009*b*). *Jpn. J. Appl. Phys.* **48**, 105502.

[bb19] Sugiyama, S., Tanabe, K., Hirose, M., Kitatani, T., Hasenaka, H., Takahashi, Y., Adachi, H., Takano, K., Murakami, S., Mori, Y., Inoue, T. & Matsumura, H. (2009*a*). *Jpn. J. Appl. Phys.* **48**, 075502.

[bb20] Tanabe, K., Hirose, M., Murai, R., Sugiyama, S., Shimizu, N., Maruyama, M., Takahashi, Y., Adachi, H., Takano, K., Murakami, S., Inoue, T., Mori, Y. & Matsumura, H. (2009). *App. Phys. Express*, **2**, 125501.

[bb21] Willaert, R., Zegers, I., Wyns, L. & Sleutel, M. (2005). *Acta Cryst.* D**61**, 1280–1288. 10.1107/S0907444905021566 16131762

